# *Youtuus*, a new bamboo-feeding genus of the tribe Augilini with two new species from China (Hemiptera, Fulgoromorpha, Caliscelidae)

**DOI:** 10.3897/zookeys.783.25135

**Published:** 2018-09-04

**Authors:** Nian Gong, Lin Yang, Xiang-Sheng Chen

**Affiliations:** 1 Institute of Entomology, Guizhou University, Guiyang, Guizhou University Guiyang China; 2 Guizhou, 550025, P.R. China Guizhou University Guiyang China; 3 The Provincial Special Key Laboratory for Development and Utilization of Insect Resources, Guizhou University, Guiyang, Guizhou, 550025, P.R. China Guizhou University Guiyang China

**Keywords:** bamboo, Caliscelini, distribution, planthopper, southern China, taxonomy

## Abstract

A new bamboo-feeding planthopper genus *Youtuus* Chen & Gong, **gen. n.** with two new species *Y.erythrus* Gong, Yang & Chen, **sp. n.** and *Y.strigatus* Gong, Yang & Chen, **sp. n.** (Hemiptera: Fulgoromorpha: Caliscelidae: Ommatidiotinae: Augilini), are described and illustrated from China. Keys to the genera of Augilini and the species of *Youtuus* Chen & Gong, **gen. n.** are given.

## Introduction

The planthopper tribe Augilini was erected by Baker (1915) in the subfamily Augilinae of family Issidae. The tribe was subsequently transferred to the family Lophopidae (Muir 1930). Later, Fennah (1987) accommodated the group as a subtribe of the tribe Ommatidiotini (Issidae: Caliscelinae). Emeljanov (1999) suggested treating Augilini as a tribe of subfamily Ommatidiotinae of family Caliscelidae based on external morphological characters including ovipositor structure which was confirmed by Gnezdilov (2003).

Modern fauna of the tribe Augilini comprises 13 genera with 27 species, known from the Oriental and Afrotropical regions (Gnezdilov and Bourgoin 2009; Gnezdilov 2013; Chen et al. 2014; Bourgoin et al. 2015). Twelve species within four genera have been reported from mainland China (Che et al. 2009; Chen et al. 2014; Yang and Chen 2014). The members of the tribe Augilini are characterized by forewing with clavus relatively long, hindwing well developed; abdomen elongate, narrowly cylindrical, with anterior and posterior margins of terga and sterna respectively transverse and chevron-like.

In this paper, a new genus with two new species of the tribe Augilini is established. Type specimens of these two species were collected from bamboo in southwestern China (Guizhou Province). The descriptions and illustrations are given. Keys to genera of Augilini and to species of the new genus are provided.

## Materials and methods

Terminology follows Fennah (1987) and Chan and Yang (1994). Dry specimens were used for the descriptions and illustrations. External morphology was observed under a stereoscopic microscope and characters were measured with an ocular micrometer. Measurements were given in millimeters; body length was measured from the apex of the head to the apex of the forewing in repose. The genital segments of the examined specimens were macerated in 10% NaOH, washed in water, and transferred to glycerin. Illustrations of the specimens were made with a Leica MZ 12.5 stereomicroscope. Photographs were taken with KEYENCE VHX-1000 system. Illustrations were scanned with CanoScan LiDE 200 and imported into Adobe Photoshop CS7 for labelling and plate composition.

The type specimens and material examined are deposited in the Institute of Entomology, Guizhou University, Guiyang, China (**IEGU**).

## Taxonomy

### Key to the genera of the tribe Augilini (modified from Fennah 1987 and Che et al. 2009)

**Table:** 

1	Frons with two or three carinae; apical segment of rostrum with distinctly narrower than long	**2**
–	Frons without carinae; apical segment of rostrum with width at least broader than long	**12**
2	Frons bicarinate	**3**
–	Frons tricarinate	**5**
3	Vertex transverse (Distant 1916: fig. 64)	*** Tubilustrium ***
–	Vertex strongly or slightly produced	**4**
4	Vertex strongly produced anteriorly; forewing with strongly sinuate costal margin (Fennah 1987: figs 10–13)	*** Symplanodes ***
–	Vertex slightly produced anteriorly; forewing with weakly sinuate costal margin (Melichar 1914: fig. 8)	*** Augilina ***
5	Vertex with anterior margin strongly produced	**6**
–	Vertex with anterior margin not or slightly produced	**8**
6	Forewing without nodal vein (Gnezdilov 2011: fig. 3)	*** Signoreta ***
–	Forewing with nodal vein	**7**
7	Fore femora and tibiae weakly dilated and strongly flattened	*** Cicimora ***
–	Fore femora and tibiae not dilated neither flattened (Chen et al. 2014: figs 2–97, 98, 99)	*** Symplana ***
8	Forewing narrowing apically (Gnezdilov 2011: fig. 1)	*** Cano ***
–	Forewing widely rounded apically	**9**
9	Vertex as long as, or longer than wide (Fennah 1987: fig. 1)	*** Symplanella ***
–	Vertex wider than long	**10**
10	Male anal segment long, lateral margin with a verruciform process (Figs 15, 27)	***Youtuus* gen. n.**
–	Male anal segment short, lateral margin without any verruciform process	**11**
11	Male with U-shaped aedeagus (Che et al. 2009: fig. 15)	*** Pseudosymplanella ***
–	Male with stick-shaped aedeagus (Emeljanov 2013: fig. 11)	*** Anthracidium ***
12	Apex of head acute in profile; frons narrow without dorsal flagellum	*** Augila ***
–	Apex of head rounded in profile; frons broad with a dorsal flagellum	**13**
13	Fore femora and tibiae distinctly dilated and flattened, femora narrower than tibiae (Emeljanov 2013: fig. 1)	*** Augilodes ***
–	Fore femora dilated and flattened, tibiae not dilated neither flattened, femora distinctly wider than tibiae (Emeljanov 2013: fig. 2)	*** Discote ***

#### Youtuus

Taxon classificationAnimaliaHemipteraCaliscelidae

Chen & Gong gen. n.

http://zoobank.org/4BA14363-0632-48F2-816C-9D35648F4303

Figs 1–8 , 9–20 , 21–32

##### Type species.

*Youtuuserythrus* Gong, Yang & Chen, sp. n.

**Diagnosis. *Description.*** Head (Figs 9, 21) with eyes as wide as pronotum. Vertex with anterior margin slightly convex, posterior margin slightly concave, lateral margins subparallel, disc depressed. Frons (Figs 10, 22) with median and lateral carinae distinct, sublateral carinae complete or basal 1/2 obsolete, widest at level of second segment of antennae. Clypeus (Figs 10, 22) with lateral carinae distinct. Pronotum (Figs 9, 21) with anterior margin roundly convex, posterior margin broadly concave, with two lateral carinae, disc with two pits slightly sank. Mesonotum (Figs 9, 21) with median carina obscure, lateral carinae weak and subparallel. Forewing (Figs 12, 24) subhyaline, relatively narrow, parallel-sided; veins distinct, claval suture present. Hindwing (Figs 13, 25) hyaline, with three lobes, longer than half length of forewing. Legs relatively long, hind tibia with a single lateral tooth; spinal formula of hind leg 8–2–2. Abdomen elongate, narrowly cylindrical, with anterior and posterior margins of terga and sterna respectively transverse and chevron-like.

***Male genitalia.*** Anal segment (Figs 14–15, 26–27) with lateral margin bearing verruciform process, ventral margin with its apical third with a row of micro brush-like bristles and 8–14 large bristles apically. Pygofer in lateral view (Figs 15, 27) with dorsal half much narrower than ventral half. Genital style (Figs 15, 18, 27, 30) longer than width. Penis (Figs 15, 19–20, 27, 31–32) extending beyond anterior margin of pygofer basally, with phallobase degenerated and obviously membranous; in lateral view with a ring structure near base of phallobase, thence apically branched one longer median and two shorter lateral processes respectively, the median one with apical half ventrally reflexed, directed basally, apex bent.

##### Remarks.

The new genus seems very closely related to *Pseudosymplanella*, but can be distinguished from the latter by: 1) frons not visible in dorsal view (frons visible in dorsal view in *Pseudosymplanella*); 2) male anal segment with lateral margin with a verruciform process, ventral margin with apical third bearing a row of micro brush-like bristles and 8–14 large bristles apically (without verruciform process and any bristles in *Pseudosymplanella*); 3) posterior margin of male pygofer in lateral view without a spine-like process (with a spine-like process in *Pseudosymplanella*). According to the structute and venation of hind wing the new genus close to Madagascan *Cano* Gnezdilov, 2011.

##### Etymology.

The name is derived from transliteration of the Chinese “you-tu”, meaning the anal segment with verruciform processes. It is masculine in gender.

##### Host plant.

Bamboo (Figs 33–34).

##### Distribution.

Southwestern China (Guizhou).

### Key to species of genus *Youtuus*

**Table:** 

1	Body mainly orange-red to red (Figs 1–4); male pygofer in posterior view without pair of processes (Figure 16)	***Y.erythrus* sp. n.**
–	Body mainly brown to dark brown (Figs 5–8); male pygofer in posterior view with pair of processes (Figure 28)	***Y.strigatus* sp. n.**

#### Youtuus erythrus

Taxon classificationAnimaliaHemipteraCaliscelidae

Gong, Yang & Chen sp. n.

http://zoobank.org/747E62F0-8A61-4248-AB67-286AC4F61E2C

Figs 1–4 , 9–20

##### Measurements.

Body length including forewing: male 5.7–5.9 mm (N = 3), female 6.2–6.6 mm (N = 3); forewing length: male 4.3–4.8 mm (N = 3), female 4.8–5.3 mm (N = 3).

##### Diagnosis.

**Figures 1–8.**
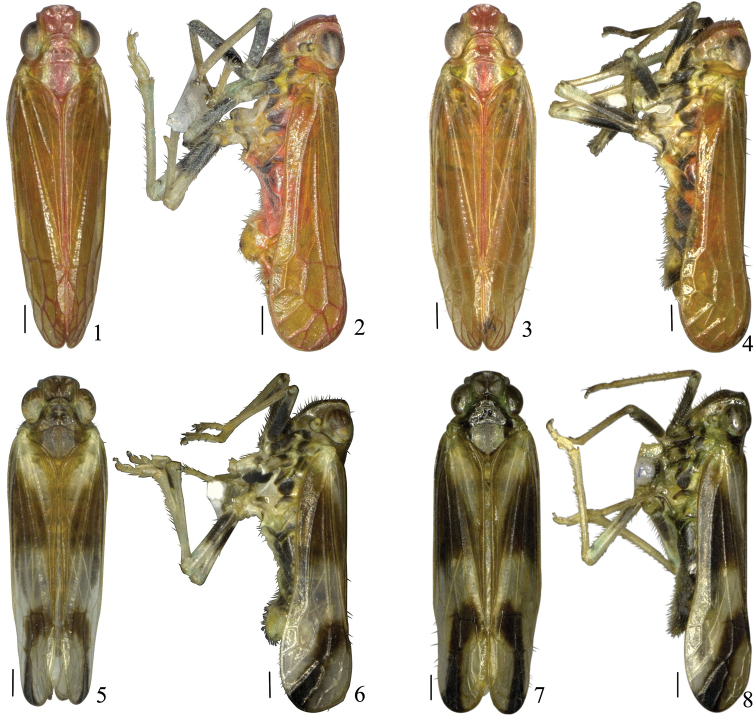
*Youtuuserythrus* Gong, Yang & Chen, sp. n. **1** Male habitus, dorsal view **2** Male habitus, lateral view **3** Female habitus, dorsal view **4** Female habitus, lateral view; *Youtuusstrigatus* Gong, Yang & Chen, sp. n. **5** Male habitus, dorsal view **6** Male habitus, lateral view **7** Female habitus, dorsal view **8** Female habitus, lateral view. Scale bars: 0.5 mm.

**Figures 9–20.**
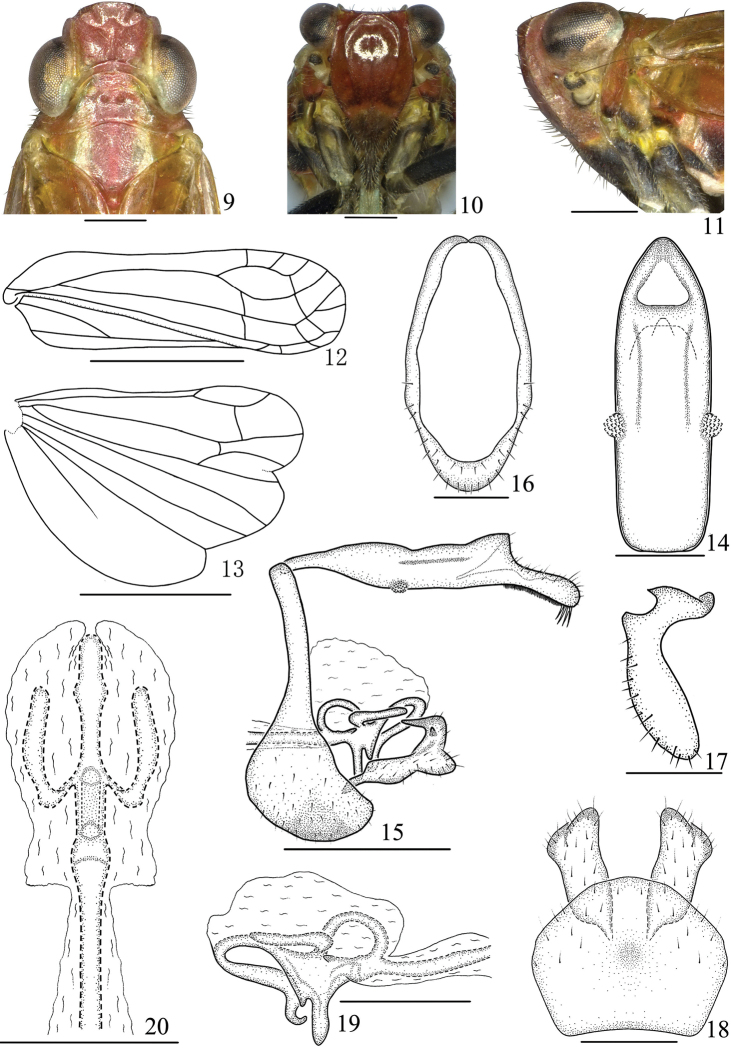
*Youtuuserythrus* Gong, Yang & Chen, sp. n., male **9** Head and thorax, dorsal view **10** Face **11** Head and thorax, lateral view **12** Forewing **13** Hindwing **14** Anal segment, dorsal view **15** Male genitalia, lateral view **16** Pygofer, posterior view **17** Genital styles, posterior view **18** Pygofer and genital styles, ventral view **19** Aedeagus, lateral view **20** Aedeagus, dorsal view. Scale bars: 2 mm (**12–13**), 1 mm (**15**), 0.5 mm (**9–11, 14, 16, 18–20**).

***Description.****Coloration*. Body mainly orange-red to red (Figs 1–4, 9–11). Ocelli reddish brown, eyes black brown (Figs 9–11). Second segment of antenna with a black transverse spot near apex (Figs 10–11). Clypeus brown (Figs 10–11). Forewing subhyaline, veins red (Figs 1–4). Hindwing hyaline, veins orange-red. Procoxae and mesocoxae dark brown, others light brown; hind legs with basal half of postcoxae dark brown, others pale yellow (Figs 1–4). Abdominal sternites with lateral margins fuscous (Figs 2, 4).

*Head and thorax*. Width of vertex (Figure 9) including eyes as wide as pronotum. Vertex (Figure 9) shorter in middle line than broad at base (0.8:1). Frons (Figure 10) 1.1 times longer in middle line than widest part. Pronotum (Figure 9) shorter in middle line than vertex (1:1.2). Mesonotum (Figure 9) 0.8 times as long as vertex and pronotum together in middle line. Forewing (Figure 12) with length 3.4 times than broad at widest part, ScP with three branches, RP single, M and CuA respectively forked in two branches apically, Pcu uniting A_1_ at basal 2/5 of clavus. Hindwing (Figure 13) 1.5 times as long as broad at widest part, ScP, RP and M single, CuA with two branches.

*Male genitalia*. Anal segment in dorsal view (Figure 14) with length 2.7 times as long as widest part; in lateral view (Figure 15) slender with dorsal margin sinuate, apically broadening to apical third, thence abruptly narrowed, lateral margin with verruciform process at basal third. Pygofer in lateral view (Figure 15) with dorsal margin distinctly shorter than ventral margin, upper half narrow, lower half wide, in posterior view (Figure 16) nearly oval, with length 2.0 times as long as widest part; in ventral view (Figure 18) with posterior margin roundly convex, anterior margin slightly concave. Genital style in lateral view (Figure 15) nearly hook-like, outer surface with a small tooth process, apex sharp, directed basad; in ventral view (Figure 18) nearly rectangle, with apex widest; in posterior view (Figure 17) with dorsal 1/3 avicular, ventral 2/3 clavate. Penis in lateral view (Figure 19) with a round ring structure near base of phallobase, a tooth-like process located at the ring inner-ventral margin, aedeagus with apex hook-shaped. Connective (Figure 19) straight and stub, fused with base of aedeagus, near its apical side with a short tooth process at base.

##### Type material.

Holotype: ♂, **China**: Guizhou Province, Xishui County, Donghuang (28°33'N, 106°20'E), on bamboo, 27 September 2017, Bin Yan; paratypes: 10 ♂♂, 17♀♀, data same as holotype, Hong-Li He and Nian Gong.

##### Host plant.

Bamboo (*Phyllostachys* Sieb. et Zucc.) (Figure 33).

##### Distribution.

Southwestern China (Guizhou).

##### Etymology.

The specific name is derived from the Latin words “*erythros*”, referring to the color of the frons.

##### Remarks.

This new species is closely related to *Y.strigatus* Gong, Yang & Chen, sp. n., but differs in: 1) body mainly orange-red to red (brown to dark brown in *strigatus*); 2) male pygofer in posterior view without pair of processes (with pair of processes in *strigatus*); 3) lateral margin of anal segment bearing verruciform process at basal third (at basal half in *strigatus*).

#### Youtuus strigatus

Taxon classificationAnimaliaHemipteraCaliscelidae

Gong, Yang & Chen sp. n.

http://zoobank.org/BCAC22D6-5C4F-4482-B3F3-EED44157CDE9

Figs 5–8 , 21–32

##### Measurements.

Body length including forewing: male 5.6–5.8 mm (N = 3), female 6.9–7.1 mm (N = 3); forewing length: male 4.8–5.1 mm (N = 3), female 5.6–5.7 mm (N = 3).

##### Diagnosis.

**Figures 21–32.**
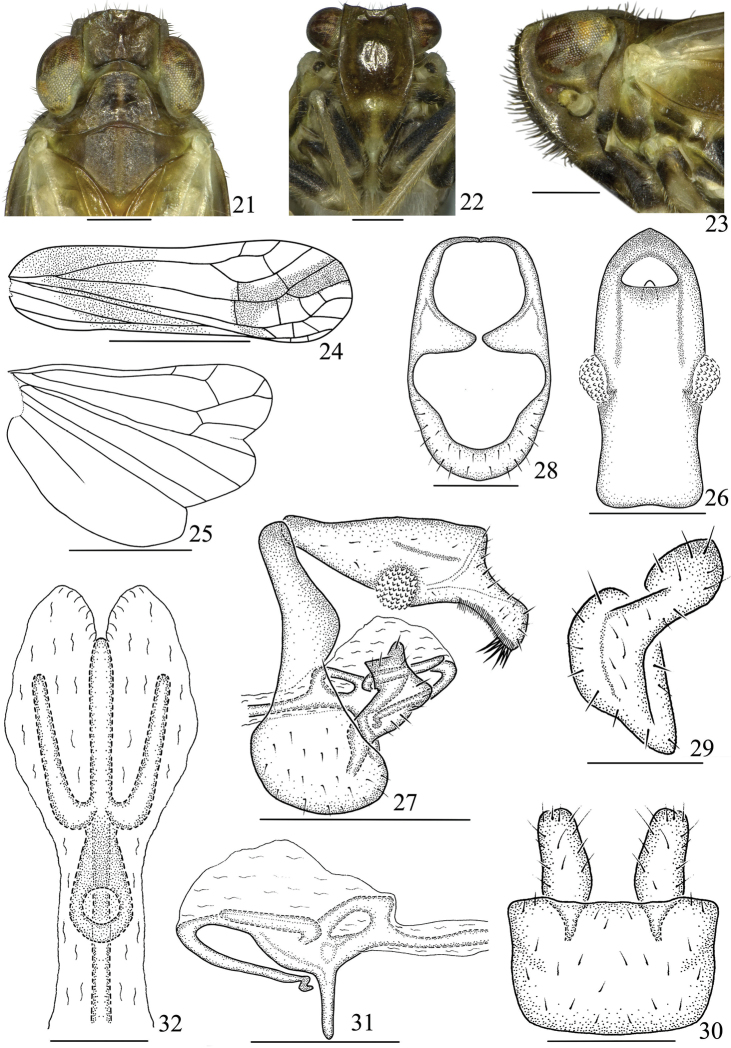
*Youtuusstrigatus* Gong, Yang & Chen, sp. n., male **21** Head and thorax, dorsal view **22** Face **23** Head and thorax, lateral view **24** Forewing **25** Hindwing **26** Anal segment, dorsal view **27** Male genitalia, lateral view **28** Pygofer, posterior view **29** Genital styles, posterior view **30** Pygofer and genital styles, ventral view **31** Aedeagus, lateral view **32** Aedeagus, dorsal view. Scale bars: 2 mm (**24–25**), 1 mm (**27**), 0.5 mm (**21–23, 26, 28, 30, 32**).

***Description.****Coloration*. Body mainly brown to dark brown (Figs 5–8, 21–23). Ocelli reddish brown, eyes black brown (Figs 21–23). Second segment of antenna with a black transverse spot near apex (Figs 22–23). Clypeus with the base and apex pale yellowish brown (Figs 22–23). Pronotum and mesonotum with areas along middle line pale yellow (Figure 21). Forewing grayish white, subhyaline, with a large dark brown transverse stripe from base of anterior margin to middle of posterior margin and a narrow dark brown longitudinal stripe from apical third to apical margin (Figs 5–8). Hindwing hyaline, veins brown. Procoxae and mesocoxae with basal half dark brown, apical half light brown; hind legs with basal half of postcoxae dark brown, others pale yellow (Figs 6, 8). Abdominal sternites yellowish brown with lateral margins fuscous (Figs 6, 8).

*Head and thorax*. Width of vertex (Figure 21) including eyes as wide as pronotum. Vertex (Figure 21) shorter in middle line than broad at base (0.7:1). Frons (Figure 22) 1.2 times longer in middle line than widest part. Pronotum (Figure 21) as long in middle line as vertex. Mesonotum (Figure 21) 0.7 times as long as vertex and pronotum together in middle line. Forewing (Figure 24) 3.4 times as long as broad at widest part, ScP with two branches apically, RP single, M and CuA respectively forked in two branches apically, Pcu uniting A_1_ at basal 1/2 of clavus. Hindwing (Figure 25) 1.5 times as long as broad at widest part, ScP and RP single, M and CuA with two branches.

*Male genitalia*. Anal segment in dorsal view (Figure 26) with length 2.1 times as long as widest part; in lateral view (Figure 27) dorsal margin slightly convex, apically broadening to apical half widest, apical third abruptly narrowed, lateral margin with verruciform process at basal half. Pygofer in lateral view (Figure 27) with dorsal margin distinctly shorter than ventral margin, upper half narrow, lower half round, posterior margin obviously convex at upper third; in posterior view (Figure 28) nearly oval, with length 1.7 times longer in middle line than widest part, a pair of large tooth-like processes located above the middle of lateral margin, point to each other; in ventral view (Figure 30) subquadrate. Genital style in lateral view (Figure 27) with apical margin broadly concave, dorsal margin with apical third dorsally uplifted and branched into two stubbed processes apically, the basal one with apical margin angularly convex, the apical one with apical margin roundly convex; in ventral view (Figure 30) nearly rectangle, with basal third widest; in posterior view (Figure 29) with base disciform, apex swollen, tongue-shaped. Penis in lateral view (Figure 31) near base of phallobase with an irregular ring structure, of which base angularly convex, aedeagus with apex S-shaped. Connective in lateral view (Figure 31) straight and slender, fused with base of aedeagus.

##### Type material.

Holotype: ♂, **China**: Guizhou Province, Suiyang County, Kuankuoshui National Natural Reserve (28°14'N, 107°00'E), on bamboo, 13 July 2017, Ya-Lin Yao; paratypes: 4 ♂♂, 6 ♀♀, data same as holotype, Nian Gong, Yong-Jin Sui and Yan Zhi; 2♂♂, 5♀♀, China: Guizhou, Duyun City, Doupengshan (26°15'N, 107°31'E), on bamboo, 9 June 2017, Liang-Jing Yang and Ya-Lin Yao.

##### Host plant.

Bamboo (*Chimonobambusa* Makino) (Figure 34).

##### Distribution.

Southwestern China (Guizhou).

##### Etymology.

The specific name is derived from the Latin words “*striga*”, referring to its color of the forewing.

**Figure 33.**
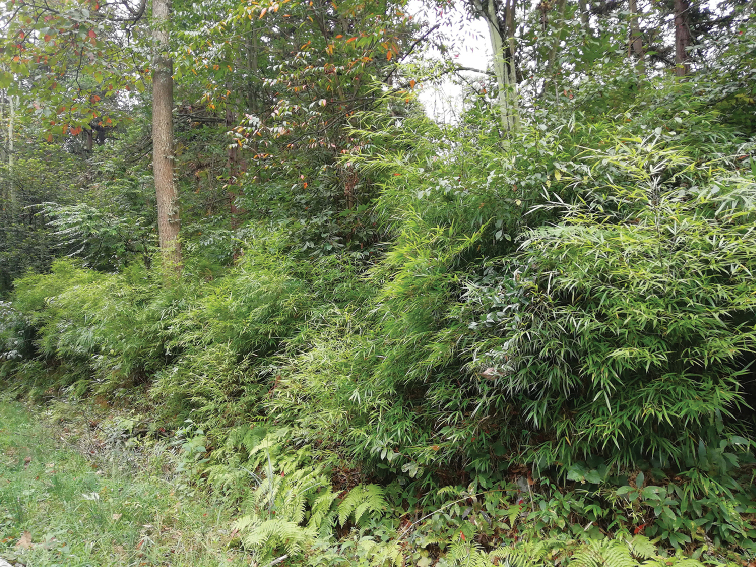
The habitat of *Youtuuserythrus* sp. n. (27 September 2017, Xishui County, photograph by Nian Gong).

**Figure 34.**
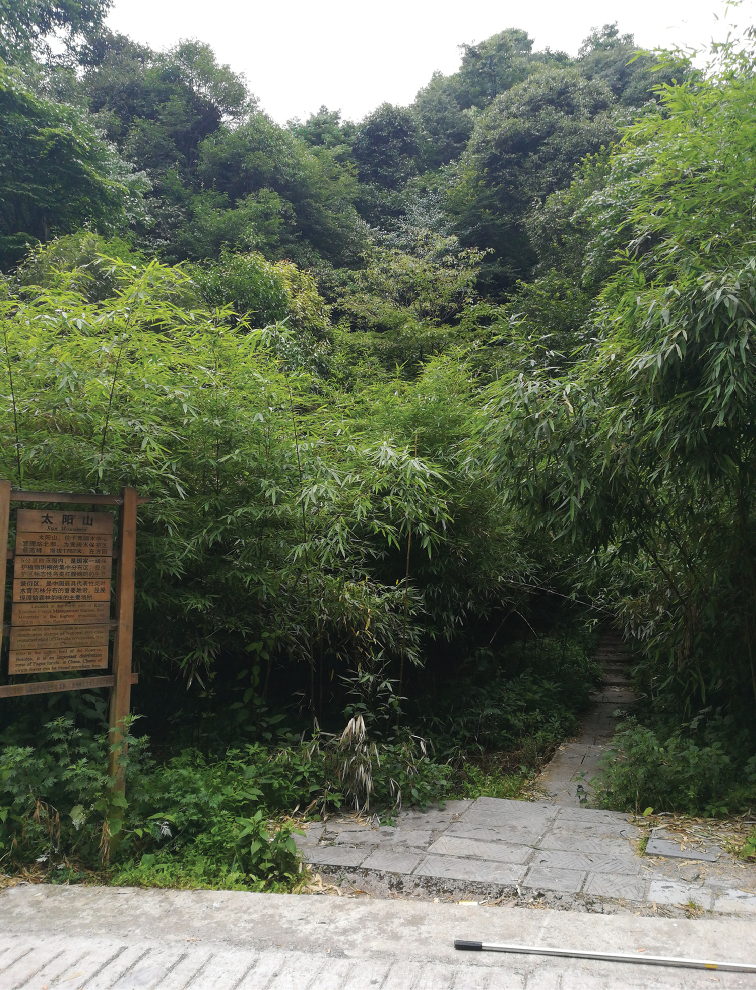
The habitat of *Youtuusstrigatus* sp. n. (13 July 2017, Suiyang County, photograph by Ya-lin Yao).

## Supplementary Material

XML Treatment for Youtuus

XML Treatment for Youtuus erythrus

XML Treatment for Youtuus strigatus
